# Supplementary material for: Is the burden of anaemia among Indian adolescent women increasing? Evidence from Indian Demographic and Health Surveys (2015–21)
Source: PLOS Glob Public Health. 2023 Sep 6;3(9):e0002117. doi: 10.1371/journal.pgph.0002117 (PMC10482272; doi:10.1371/journal.pgph.0002117)
Supplement: S1 Table — (DOCX) [file pgph.0002117.s001.docx]

**S1 Table: Variance inflation factors**

| **Background characteristics** | **VIF** | **1/VIF** |
| --- | --- | --- |
| **Biodemographic and socioeconomic factors** | |  |
| **Marital status** |  |  |
| Mot married (ref) |  |  |
| Married before 18 years | 1.64 | 0.61 |
| Married on 18 years and above | 1.12 | 0.89 |
| **Parity** |  |  |
| No child (ref) |  |  |
| Single child | 1.94 | 0.51 |
| 2 and more children | 1.22 | 0.82 |
| **Level of education** |  |  |
| No education (ref) |  |  |
| Primary | 1.99 | 0.50 |
| Secondary | 3.14 | 0.32 |
| Higher | 2.20 | 0.45 |
| **Social groups** |  |  |
| SC (ref) |  |  |
| ST | 1.97 | 0.51 |
| OBC | 1.90 | 0.53 |
| Others | 1.74 | 0.58 |
| **Religion** |  |  |
| Hindu (ref) |  |  |
| Muslim | 1.22 | 0.82 |
| Christian | 1.80 | 0.55 |
| Others | 1.12 | 0.89 |
| **Household wealth index** |  |  |
| Poorest (ref) |  |  |
| Poorer | 1.75 | 0.57 |
| Middle | 1.95 | 0.51 |
| Richer | 2.13 | 0.47 |
| Richest | 2.33 | 0.43 |
| **Geographical factors** |  |  |
| **Place of residence** |  |  |
| Urban (ref) |  |  |
| Rural | 1.36 | 0.73 |
| **Region of residence** |  |  |
| North (ref) |  |  |
| Central | 2.04 | 0.49 |
| East | 2.07 | 0.48 |
| West | 1.36 | 0.73 |
| Southern | 1.70 | 0.59 |
| North-east | 2.36 | 0.42 |
| **Behavioural factors** |  |  |
| **Mass Media exposure** |  |  |
| No (ref) |  |  |
| Low | 2.25 | 0.44 |
| Medium | 2.51 | 0.40 |
| High | 1.25 | 0.80 |
| **Dietary habit** |  |  |
| Vegetarian (ref) |  |  |
| Non-vegetarian | 1.39 | 0.72 |
| **Alcohol consumption** |  |  |
| No (ref) |  |  |
| Yes | 1.04 | 0.96 |
| **Consumption of tobacco in any form** |  |  |
| No tobacco (ref) |  |  |
| Uses tobacco: smoke or smokeless | 1.07 | 0.93 |
| **Current contraceptive use** |  |  |
| Not using (ref) |  |  |
| Traditional method | 1.08 | 0.93 |
| Modern method | 1.14 | 0.88 |
| **Health related factors** |  |  |
| **Body Mass Index** |  |  |
| Underweight (ref) |  |  |
| Normal weight | 1.10 | 0.91 |
| Overweight | 1.07 | 0.93 |
| Obese | 1.02 | 0.98 |
| **Have diabetes** |  |  |
| No (ref) |  |  |
| Yes | 1.00 | 1.00 |
| **Currently amenorrhoeic** |  |  |
| No (ref) |  |  |
| Yes | 1.48 | 0.68 |
| **Mean VIF** | **1.63** |  |

Note: ref= reference category
