# Supplementary material for: Is the burden of anaemia among Indian adolescent women increasing? Evidence from Indian Demographic and Health Surveys (2015–21)
Source: PLOS Glob Public Health. 2023 Sep 6;3(9):e0002117. doi: 10.1371/journal.pgph.0002117 (PMC10482272; doi:10.1371/journal.pgph.0002117)
Supplement: Attachment — Submitted filename: 230606_Plos GPH Response to Reviewers R1.docx [file pgph.0002117.s002.docx]

*Dear Dr Rajesh Sharma,*

*We want to thank you for providing us with an opportunity to revise our paper entitled* ***“Is the burden of anaemia among Indian adolescent women increasing? Evidence from Indian demographic and health surveys (2015-21)” (PGPH-D-23-00195)****. We would also extend our heartfelt thanks to reviewers for their valuable time and helpful comments and suggestions. The manuscript has been revised in light of the concerns raised in the review report. We have addressed all the issues raised and incorporated all the comments and suggestions made by the reviewers. Our point-to-point response to the comments, on which the reviewers needed further clarification from us or suggested modifications in the manuscript, is given below.*

**REVIEWER(S)’ COMMENTS TO THE AUTHOR**

**Reviewer 1**

Review of ***“Is the burden of anaemia among Indian adolescent women increasing? Evidence from Indian demographic and health surveys (2015-21)***” Thank you for the opportunity to review this informative manuscript. The manuscript describes a study that focuses on the prevalence of anaemia among adolescents in India using two demographic health surveys from 2015 and 2021. The study’s goals were to measure the change in the overall prevalence in the country and to explore factors that may influence anaemia rates. The main findings include the increased anaemia prevalence and several risk and protective factors. The authors provide their interpretations of the overall and specific findings and suggest policy directions toward better control of anaemia.

This manuscript deals with one of the most important and persistent global public health problems. Anaemia has drawn so much public health efforts over decades. The finding that anaemia is on the rise among adolescents in nationally representative samples has startling implications for India and many other countries. As such, this manuscript has exciting potential to become an important contribution to PLOS Global Public Health. Having said this, this manuscript is underdeveloped in several regards, including the conceptual framework, analytic approach, and presentation and discussion of results.

**Main comments**

**Comment#1:** I would like to see a careful revision on the definition of anaemia. The paper focuses on anaemia of low hemoglobin concentration, rather than “deficient” hemoglobin, which implies hemoglobin polymorphisms like sickle-cell trait, which can also result in anaemia. You do mention the importance of iron and other micronutrients and (to a lesser extent) infection. So, it is implicit that this study is concerned with anaemia of diverse types. I feel that the manuscript could be strengthened by paying more attention to anaemia arising from infection. Low hemoglobin could occur as part of immune response to infections or disease processes. Even though nutritional (particularly iron deficiency) anaemia is usually the most prevalent, other types of anaemia are also of crucial importance. This is particularly the case when one tries to understand why preventive programs focused on iron and folate supplementation have had low efficacy. Anaemia of infection (either as a genetic adaptation to malaria or phenotypic response to combat infections) should be incorporated in the introduction and discussion, and ideally in method and analysis as well. It is my understanding that recent Demographic Health Survey data often include biomarkers of infection/inflammation. It might even be possible to include it in the statistical evaluation.

**Response#1:** Thank you for your suggestion, sir. We appreciate your insight that anaemia of infection should be considered in our study and we are open to exploring this avenue. However, upon further investigation, we discovered that the National Family Health Survey (NFHS) only assesses anaemia status through hemoglobin concentrations using capillary blood samples, and does not include biomarkers for infection or inflammation. Therefore, we are unable to include biomarkers of infection/inflammation in our current study. To avoid confusion, we have made it clear in the introductory text that our study is solely focused on anaemia resulting from low hemoglobin concentrations. You can find the modified text making this clear in lines- 54-63.

**Comment#2:** For the conceptual framework, it seems inadequate to place health-related factors entirely separated from geographical/demographic factors. It seems like a better idea to think of geographical and economic factors as more distal factors that may influence some health factors. One could then construct the framework where economic factors have direct and indirect effects on anaemia prevalence, the latter mediated by health factors. I understand that it is necessary to simplify concepts for clarity, but treating geographical and demographic factors completely separately from health factors is unrealistic and prevents this study from addressing the interplay between factors that the authors attempt to highlight.

**Response#2**: Thank you for your insightful comment on the conceptual framework presented in the study. Following your suggestion, we have modified the framework to reflect the possibility of health-related factors having a direct effect on anaemia prevalence, while acknowledging the indirect influence of geographical and socioeconomic factors on health-related factors. We hope that the modified framework provides a more comprehensive understanding of the determinants of anaemia**. [Please see Figure 2].**

Please see below my specific comments and suggestions.

**Detailed comments**

**Abstract**
**Comment#3:** “Despite several government initiatives…. The burden of anaemia has increased over time” - I do not recall seeing comparable statements and references in the introduction section.

**Response#3:** Thank you for pointing this out. As the above statement was out of context in our abstract, we have removed the sentence from our revised manuscript.

**Comment#4:** “Factors such as nutrition, parity, …. Have a role in the prevalence of anaemia.” – The word “role” implies a causal influence on anaemia. Please consider editing appropriately for an association study.

**Response#4:** Thank you for your suggestion. As per your comment, we have replaced the term “role” with appropriate word.

The revised text now reads – “*Several factors were found* ***associated*** *with anaemia, including having more than one child (AOR: 1.33, 99% CI: 1.16-1.51), having no education (AOR: 1.25, 99% CI: 1.16-1.34), belonging to Scheduled Tribes (AOR: 1.47, 99% CI: 1.40-1.53), being in the lowest wealth quintile (AOR: 1.17, 99% CI: 1.12-1.23), year of survey (AOR: 1.26, 99% CI: 1.23-1.29), and being underweight (AOR: 1.10, 99% CI: 1.07-1.12).”* **[lines 45-48]**

**Introduction
Comment#5:** Line 47 “a deficiency in red blood cells or haemoglobin” does not do justice to the definition of anaemia more broadly.

**Response#5:** Thank you for pointing this out. We have now replaced the statement “Anaemia is a common condition characterized by a deficiency in red blood cells or haemoglobin, the protein that carries oxygen in the blood” with the World Health Organization’s definition of Anaemia.

The revised text now reads: “*According to the World Health Organization (WHO), anaemia is a disorder in which the number of red blood cells or haemoglobin concentration within the red blood cells is below normal which subsequently results in the decreased oxygen-carrying capacity of blood [1].*” **[lines 54-56]** and “*WHO defines anaemia in pregnant women as a haemoglobin concentration <11.0 g/dl, and anaemia in non-pregnant women as a haemoglobin concentration <12.0 g/dl [2]*” **[lines 61-63]**, in our revised manuscript.

This definition has been taken from the WHO website for which link is given below. WHO. The Global Prevalence of Anaemia in 2011. Geneva World Heal Organ. 2015. Available: <https://apps.who.int/iris/bitstream/handle/10665/177094/9789241564960_eng.pdf>

**Comment#6:** Line 50 Please define “GBD.” Also, this sentence implies that anaemia directly kills. Is it true? Or is anaemia attributed to a distal cause for things like infectious diseases that kill people? Either way, clarifying this with sources would be important.

**Response#6:** Thank you for highlighting this important issue. We agree that anaemia does not directly kill, rather anaemia is mainly responsible for millions of years of healthy life lost due to disability (YLDs). In our modified manuscript, we have modified the text in **lines 64-66**, including the word GBD, and have provided suitable sources in support of our statement. We have now clearly written- “*Globally anaemia is responsible for approximately 50.3 million years of healthy life lost due to disability (YLDs) in 2019 with highest YLDs attributable to anaemia coming from South Asia, Western Sub-Saharan Africa and Eastern Sub-Saharan Africa”.*

**Comment#7:** Line 59 Parasitic infections are mentioned here, but it does not make clear the multiple roles infectious diseases can play in malaria prevalence. Anaemia due to parasitic infections (e.g., hookworm) and anaemia due to the human body’s immune response (i.e., iron sequestration from infectious agents) are important causes of low circulating hemoglobin in environments of high infectious load. As currently written, this section gives the impression that anaemia is caused by malnutrition, which happens to coincide geographically with high rates of parasitic infections. This minimizes the importance of infections as a direct cause of anaemia. (Although it is also true that malnutrition increases the risk for infectious diseases).

**Response#7:** Thank you for your feedback. We have revised our manuscript and removed the mention of parasitic infections to avoid creating a misleading impression about the causes of anaemia. While we acknowledge the role of infectious diseases, including parasitic infections, in anaemia prevalence, our study does not specifically address these multiple roles. We appreciate your insight on the complex relationship between infections and anaemia.

**Comment#8:** Line 100 It is good to see the reference to the idea, “infections that can cause anaemia” here, but the paper would be strengthened by increased attention to this type of anaemia.

**Response#8:** Thank you for your feedback. We understand the importance of addressing anaemia caused by infections in the context of our research. However, the dataset we utilized for our study, the NFHS dataset, unfortunately does not provide any information about infections among women. Therefore, we were unable to explore this aspect in our analysis. We have taken your suggestion into consideration and included a statement in the limitation section of our revised manuscript, acknowledging the absence of information on infections and its potential association with anaemia.

The limitation section of the revised manuscript now states: "*Additionally, anaemia can have a significant association with different types of infections. However, since NFHS does not provide any information about infections among women we could not address this issue*" **(lines 492-494).**

We appreciate your input and have made the necessary revisions to improve the clarity of our paper.

**Comment#9:** Line 118 “poverty which limits access to nutritious food and poor sanitation …” does not seem to make sense. Please edit.

**Response#9:** Thank you for pointing out the issue with the sentence. We appreciate your feedback and have revised it accordingly. The sentence now reads: " *Evidence suggests that poverty, which limits individuals access to nutritious food and healthcare, is a major determinant of anaemia these countries [33]. " (****Lines 103-105****).* We have made the necessary edits to improve the clarity and coherence of the sentence.

**Comment#10:** The paragraph starting with line 125 seems to include statements that require citation of sources. Line 128, the notion that societal views that prioritize men over women leads to women’s inadequate nutritional consumption should be supported by sources, even though this makes intuitive sense.

**Response#10:** We appreciate your feedback regarding the need for proper citation of sources in the paragraph starting with line 125 and 128. We have added appropriate citations to both sentences in question. Thank you for bringing this to our attention.

**Comment#11:** Line 131. Please cite sources for the notion that anaemia increases due to iron loss via breast milk. This is contrary to my understanding that human milk does not draw significant amounts of iron from the mother compared to the amounts drawn during pregnancy. Iron-binding proteins like lactoferrin are abundant in milk, but they seem to serve more of immune role than iron transport based on more recent research. So, the information you provide might be outdated and therefore requires either revision or proper citation to allow the reader to evaluate the information quality.

**Response#11:** We appreciate your feedback and clarification on the topic of iron loss via breast milk. Upon further research and consideration, we realised that the information provided in line 131 was outdated and not accurate.

After reviewing the current research on the topic, we have decided to remove the statement regarding anaemia increasing due to iron loss via breast milk from our revised manuscript. It is important to provide accurate and up-to-date information to the readers, and we apologize for any oversight in this regard.

Thank you for your valuable your input in ensuring the quality and accuracy of our paper.

**Comment#12:** Paragraph starting with 139. This paragraph would be strengthened if you added hypotheses or expectations, or specific aims. Currently, some of this seems to appear in your data and method section (e.g., independent variables), but it makes better sense to state your aims or hypotheses first and then you describe the methods you used to accomplish those aims.

**Response#12**: Thank you for your comment. We have carefully considered your suggestion and made significant revisions to the last paragraph of our introduction. We have explicitly stated our specific aims and hypotheses to provide clarity on the objectives of our study [**lines 124-131**]. Additionally, we have thoroughly edited the introduction to improve its overall quality. We appreciate your feedback, as it has helped us enhance the structure and content of our manuscript.

**Comment#13:** Line 155. I understand that the sampling strategies for surveys are available elsewhere, but it would help the reader if you provided a brief description of sampling strategies for the two surveys. Were the strategies identical across two surveys?

**Response#13:** Thank you for your suggestion. We have included a brief description of sampling strategies for the two surveys in the ‘Data source’ section of the paper [**lines 134-158**]. **Yes,** the strategies were similar across the two surveys used in the analysis.

**Comment#14:** Line 173 Please consider editing this sentence to reflect the cross-sectional research design. I am concerned about the word, “affecting” which implies causal order, which is not warranted.

**Response#14:** Thank you for your comment and suggestion regarding the sentence in question. We agree that the term "affected" might imply a causal relationship, which is not appropriate for a cross-sectional research design.

Based on your feedback, we have revised the sentence to reflect the appropriate terminology. The updated sentence now reads: "*This study has used a set of independent variables to examine the factor associated with anaemia prevalence among adolescent women in India."* **[lines 184-185]**

**Comment#15:** Line 183 Please define what “Scheduled” means.

**Response#15:** The term “Scheduled” in Scheduled Castes (SCs) and Scheduled Tribes (STs) means that these communities are ‘listed’ (or "scheduled") in various Government of India orders issued in the past (for various order or amendments acts related to SCs and STs, please check this Government of India website <https://socialjustice.gov.in/common/76750>). The Constitution of India has a provision for the protection and advancement of these socially and economically disadvantaged groups. The list is maintained by the National Commission for Scheduled Castes and the National Commission for Scheduled Tribes. Therefore, the term "Scheduled" is used to refer to these specific groups of people who are recognized and protected under the Indian Constitution.

To ensure clarity and understanding, we have expanded the description of various social groups, including SCs and STs, in **Table 1** where independent variables are defined. This expanded description will provide a more comprehensive understanding of these terms and their significance within the Indian context.

**Comment#16:** Line 184 “backword” classes – please either explain this or edit this expression. It sounds value-laden and offensive to me (from a global perspective). I realize this is something to do with the caste system, but it would be preferable to avoid discriminatory expressions if that is possible and appropriate.

**Response#16:** Thank you for bringing this concern to our attention. We understand your perspective and the importance of using inclusive and respectful language. The term "Other Backward Classes" (OBCs) is used in India to refer to social groups that have historically faced social and educational disadvantages. The term is recognized and used in official government records to address inequalities and provide support to these communities. It is important to note that the term "backward" in this context does not carry a negative connotation, but rather highlights the historical disadvantages that these groups have experienced.

While it is recognized and used in official government records for the purpose of addressing inequalities and providing support, we acknowledge that its usage may not be universally understood and can be sensitive in a global context.

To ensure clarity and sensitivity, we have taken your suggestion into consideration. In **Table 1** where variable descriptions are provided, we have included a note to clarify that the term **"backward" does not carry a value judgment**, but rather refers to the historical social and economic disadvantages experienced by these groups.

Here is the official link of Indian Government website, from where you can identify that this term ‘Other Backward Classes’ is official and recognised by Indian government, and not created by authors.

<http://www.ncbc.nic.in/user_panel/GazetteResolution.aspx?Value=mPICjsL1aLvxbegUDuc3MN4eB5E3Ecc1drRPAf1qXQ%2Bl0IqIfhjN1xHrf4i2h5g0>

**Comment#17:** Line 186 Please provide cutoffs for wealth index.

**Response#17:** The wealth index is a composite measure of a household's socio-economic status and is widely used in demographic and health research. The wealth index cutoffs are determined based on the scores assigned to households using principal component analysis. The scores are based on the number and kinds of consumer good they own, ranging from a television to a bicycle or car and housing characteristics like source of drinking water, toilet facilities, and flooring materials.

To determine the national wealth quintiles, the households are ranked based on their household scores and sorted in ascending order. The entire distribution of households is then divided into five equal parts called quintiles, with each quintile containing 20 percent of the households. The bottom 20% households are categorised as Poorest, the next as Poorer. The third quintile is called Middle. The fourth and fifth (top) quintile are called Richer and Richest.

***It is important to note that the wealth index used in our study was not created by the authors.*** ***The variable was already present in the National Family Health Survey (NFHS-5) dataset.***

**Comment#18:** Line 187 Please provide information on media exposure. Is no exposure based on self-reporting? I wonder about the validity of this category – no exposure means no radio, no TV, or no newspaper? Have you considered combining no exposure with low exposure to obtain three groups instead of four?

**Response#18:** Thank you for your comment. In response to your query, we have provided a detailed description of mass-media exposure and how it was created in Table 1 of the revised manuscript.

In the NFHS-5 survey, women were asked three questions related to their media consumption habits: 1. How often do they read newspapers/magazines? 2. How often do they watch television? 3. How often do they listen to the radio? The response options provided were: 'almost every day', 'at least once a week', 'less than once a week', and 'not at all'. These options were dichotomised into ‘yes’ or ‘no’ to determine whether the respondent read newspapers/magazines, watched television, and listened to the radio. Then combining the responses of these three dichotomous variables, we created a new variable to determine the level of mass-media exposure for each respondent. It had four categories as follows:

'No exposure' (coded as 0): This category included respondents who reported not being exposed to any form of mass media.

'Low exposure' (coded as 1): This category included respondents who reported being exposed to only one type of mass media (e.g., either newspapers/magazines, television, or radio).

'Medium exposure' (coded as 2): This category includes respondents who reported being exposed to any two types of mass media.

'High exposure' (coded as 3): This category includes respondents who reported being exposed to all three types of mass media.

Thank you for your suggestion regarding combining the categories of no exposure and low exposure. However, we respectfully disagree with this proposal as these two groups represent distinct categories. The absence of media exposure is fundamentally different from having exposure to only one form of media. Therefore, we have chosen to maintain separate categories for accurate representation and analysis.

We hope this clarification addresses your concerns. Thank you for bringing up this point, and we have made the necessary revisions to provide a clearer explanation of the mass-media exposure variable in our revised manuscript.

**Comment#19:** Line 196 You mention that you used descriptive statistics for background characteristics and anaemia prevalence by group categories. Did you evaluate associations/correlations among predictors? Please consider doing so and provide this information before presenting regression models.

**Response#19:** Thank you for your suggestion. Yes, we have evaluated the associations/correlations among predictors and checked the multicollinearity among them. The variance inflation factors are presented in a separate table (**supplementary table 1**).

**Comment#20:** Line 198 Is multivariable the same as multivariate? I am not familiar with the former expression.

**Response#20:** Although multivariable and multivariate are used interchangeably, these terms are slightly different. While multivariable model is used for the analysis with one outcome (dependent) and multiple independent (a.k.a., predictor or explanatory) variables, a multivariate model is used for the analysis with more than one outcome (dependent) and multiple independent variables. However, not many researchers are attentive to the distinction. The difference between these two terms was brought to attention by Hidalgo and Goodman in 2013^1^. For your perusal I am giving the link of the main two articles where the clear definitions of these terms are given-

1. Hidalgo, B. & Goodman, M. Multivariate or multivariable regression? Am. J. Public Health 103, 39–40 (2013) https://ajph.aphapublications.org/doi/full/10.2105/AJPH.2012.300897

2.	Kalan, M. E., Jebai, R., Zarafshan, E. & Bursac, Z. Distinction Between Two Statistical Terms: Multivariable and Multivariate Logistic Regression. Nicotine Tob. Res. 23, 1446–1447 (2021). <https://academic.oup.com/ntr/article/23/8/1446/5812038>

**Comment#21:** Also, you used binary logistic regression models, but your outcome variable is described as ordinal with a few cutoff levels of hemoglobin. Or did you code the variable dichotomously using different cutoffs for each set of models? Please clarify.

**Response#21:** Thank you for asking this important question. In the dependent variables section of our revised manuscript, we have clearly mentioned the categorization of anaemia [**lines 169-182].**

The clarification stated- “*The variable representing the level of anaemia in both the NFHS-4 and NFHS-5 datasets is an ordinal variable having four categories: no anaemia, mild anaemia, moderate anaemia, and severe anaemia. For nonpregnant women, mild anaemia is defined as hemoglobin levels between 11.0 to 11.9 g/dl, while for pregnant women it is defined as hemoglobin levels between 10.0 to 10.9 g/dl. Moderate anaemia is defined as hemoglobin levels between 8.0 to 10.9 g/dl for nonpregnant women, and 7.0 to 9.9 g/dl for pregnant women. Severe anaemia is defined as hemoglobin levels of 8.0 g/dl or less for nonpregnant women, and 7.0 g/dl or less for pregnant women. [47].*

*To analyze the association between anaemia and various factors among reproductive women, we created a binary outcome variable from the four categories of anaemia in the NFHS-5 dataset. Women with either mild, or moderate, or severe anaemia were considered “to have anaemia” and were coded as "1". Women with no anaemia were coded as "0" or "women do not have anaemia". Our main aim was to identify whether women were anaemic or not anaemic, and this binary variable helped us to categorize women into these two groups.*”

**Comment#22:** On the four models and determination to retain or drop some variables, please consider adding a brief discussion of your reasoning behind this sequence of modeling – why certain sets of variables were evaluated first and health variables last. This may be a conventional sequence in the authors’ fields, but it does not seem intuitive to interdisciplinary readers

**Response#22:** Thank you for bringing up the concern regarding the sequence of modelling and the lack of intuitive reasoning behind it for interdisciplinary readers. We acknowledge that our initial approach may not have been clear in explaining why certain sets of variables were evaluated first and health variables were assessed last. In response to this feedback, we have revisited our analysis. Instead of using four separate models, we now have a single comprehensive model that includes all potential predictors simultaneously.

**Comment#23:** Line 204 You used the statistical significance cut-off of .05 – is this level appropriate for this large sample size? Have you considered a lower cut-off such as .01 or .001? Your sample size is quite large, and that can inflate the chances of finding statistical significance. You might have already consulted with a statistician, but if you have not, I would.

**Response#23:** Thank you for your comment and advice regarding the statistical significance cut-off in relation to our large sample size. We appreciate your input and have taken it into consideration.

In light of your concern, we have re-evaluated our analysis and made adjustments to address this issue. Specifically, we have rerun the analysis using a lower significance level of 0.01 instead of the previously used cut-off of 0.05. By employing a more stringent significance level, we have tried to mitigate the potential inflation of statistically significant findings that can occur with a large sample size.

We acknowledge the importance of consulting with statisticians and experts in the field. While we have not explicitly mentioned it in the paper, we have indeed sought the advice and guidance of a statistician during the analysis and interpretation of our data. Their also advised is use significance level of 0.01 or lower.

**Comment#24:** Around here, please add a statement on how the change over time was evaluated. From the logistic model table, I am guessing that you combined the two sets of survey data to run the models. This is not forthcoming in your description of the methodological approach to statistical analysis.

**Response#24:** Thank you for this comment and suggestion. We have revised the statistical analysis section of our manuscript to include more information on how the change over time was evaluated. The updated section now clearly describes the approach we used.

In the revised 'statistical analysis' section **(Lines 211-225)** of our manuscript, we have made the following additions:

*“In the initial stage, we presented the distribution of the sample of adolescent women across various background characteristics. Following that, we calculated the prevalence of anaemia among these adolescent women and examined how it varied based on their respective background characteristics. This analysis was performed separately for both the survey periods of 2015-16 and 2019-21, enabling us to observe any changes or trends in anaemia prevalence over time within specific subgroups defined by their background characteristics.*

*To assess the temporal change in anaemia prevalence during the study period, we initially calculated the prevalence of anaemia at the national level. However, as national-level data may obscure regional variations, we proceeded to calculate the prevalence of anaemia for each individual state during both time periods. This allowed us to analyse the state-level changes in anaemia prevalence between 2015-16 and 2019-21. By examining the state-level data, we could gain insights into any variations or trends in anaemia prevalence at a more granular level.”*

**Comment#25:** Please provide information on how you evaluated model fit and issues like multicollinearity.

**Response#25:** Thank you for highlighting the importance of evaluating model fit and addressing issues such as multicollinearity in our analysis.

To address the issue of *multicollinearity*, we computed the variance inflation factor (VIF) for each predictor. The VIF helps identify the presence of multicollinearity by quantifying the extent to which a predictor variable is influenced by linear relationships with other predictors. Lower VIF values indicate less multicollinearity and enhance the reliability of our regression model.

In our analysis, we assessed the fit of the regression model by considering several indicators. Firstly, we examined the *pseudo-R-squared* value, which provides an estimate of the proportion of variance explained by the predictors in the model. A higher pseudo-R-squared value suggests a better fit between the model and the observed data. Furthermore, we conducted the *Hosmer-Lemeshow* goodness-of-fit test. This test assesses the agreement between the observed and predicted outcomes and provides a measure of how well our model fits the data. A non-significant result suggests a good fit between the model and the observed data. Additionally, we evaluated the *log likelihood*, which measures the likelihood of obtaining the observed data based on the fitted model. A lower log likelihood indicates a better fit between the model and the data. We have updated the statistical analysis section in the revised manuscript to include the abovementioned information see **lines 241-254.**

**Comment#26:** Line 216 “either breastfeeding not pregnant” does not make sense. Please edit.

**Response#26**: Thank you for pointing out this mistake. We have corrected the text. The text now reads “*Over 90% of women in both rounds were neither pregnant nor breastfeeding*” [**lines 262-263].**

**Comment#27:** Line 218 Please define SC/ST.

**Response#27:** Thank you for pointing this issue. In the revised text we have defined SC/ST and you could find a detailed description of the term SC/ST, **in table 1**, which now reads: “*The "social group" variable in our study refers to the categorization of individuals into different social groups based on the official categorization adopted by the Government of India. The four categories included in this variable are Scheduled Castes (SC) (coded as 1), Scheduled Tribes (ST) (coded as 2), Other Backward Classes (OBC), and Others (coded as 4).*

*This categorisation of Indian population into four broad social groups has evolved over time and has its roots into India’s caste system, a rigid and discriminatory social stratification system, primarily governs the majority of the population. The communities at the bottom of the caste-based social hierarchy have been subjected to centuries of discrimination, oppression, and abuse from those higher up in the hierarchy. These communities are known as Scheduled Castes. They are officially recognised in the Constitution of India and listed in various government orders. Other Backward Classes (OBCs) are also socioeconomically and educationally disadvantaged communities, but their position in the social hierarchy is slightly higher than SCs who are placed at the bottom of the caste hierarchy. The remaining caste groups, which form the hierarchy’s top order, are grouped into a residual category called ‘Others’. Scheduled Tribes (STs), on the other hand, include tribal communities which are characterised by their unique cultural practices and geographical isolation rather than their placement in the caste hierarchy. STs continue to be one of India’s most marginalised and disadvantaged communities, having faced several social, economic, and political challenges throughout history. SC, ST, and OBC communities are recognized and protected under the Indian Constitution, and the Constitution of India provides for their protection and advancement. The National Commission for Scheduled Castes, National Commission for Scheduled Tribes, and National Commission for Backward Classes maintain the list of these communities [46,47].”*

**Comment#28:** Line 224 Did you distinguish amenorrhea types (pregnancy, postpartum, undernutrition)? This would be useful, here and/or in variable descriptions.

**Response#28:** Thank you for your comment regarding the distinction of amenorrhea types (pregnancy, postpartum, undernutrition) in our analysis. We appreciate your suggestion, but unfortunately, the NFHS-5 data that we utilized for our study does not provide specific information on the types of amenorrhea. The NFHS-5 survey collects self-reported data on amenorrhea, which is primarily focused on identifying the absence of menstruation among women of reproductive age. However, it does not delve into the specific categorization of amenorrhea types such as pregnancy-related, postpartum, or due to undernutrition.

**Comment#29:** Line 230 “before the legal age of marriage” this information should come in your variable description instead of results. Also, “two or more two children” does not make sense.

**Response#29:** Thank you for this suggestion. In the revised manuscript, we have removed this phrase from the line 230 and have added it in the variable description provided in **Table 1.** Furthermore, we have corrected the error in the sentence mentioning "two or more two children" to ensure accuracy in the revised manuscript.

**Comment#30:** Line 233 I see the expression “more anaemic” here and elsewhere. It implies more severe anaemia. Please consider using expression higher or lower prevalence instead.

**Response#30**: Thank you for bringing this to our attention. We have carefully reviewed the manuscript and made the necessary changes based on your feedback. We have replaced the expression "more anaemic" with more appropriate terminology such as "higher prevalence" or "lower prevalence" to accurately convey the severity of anaemia. We appreciate your valuable input in improving the clarity and precision of our work.

**Comment#31:** Line 234 please replace “anaemia is” with “anaemia was”

**Response#31:** Thank you for pointing out the grammatical error in our manuscript. We appreciate your attention to detail. We have replaced "anaemia is" with "anaemia was" in the revised version of the manuscript.

**Comment#32:** Line 253 In describing the map, the main text seems to focus on the state-level changes (which state claimed the highest prevalence, etc.) and misses what I perceive as the most striking overall pattern of change - the expanded regions with high anaemia prevalence in 2021.

**Response#32:** Thank you for your valuable feedback. We have revised the section describing state-wise trends in anaemia prevalence to better address your concern and emphasize the expanded regions with high anaemia prevalence in 2019-21. The revised text now provides a cohesive and meaningful overview of the state-level patterns. We have provided two major sections to describe the regions with high anaemia prevalence in 2015, 2021, and state-level changes. Those sections in the modified manuscript can be found in- ***Prevalence of anaemia among adolescent women by background characteristics* [lines 275-291]** *and* ***Variation in anaemia prevalence across regions and states of India* [lines 297-313].**

**Comment#33:** Table 3 Please provide a caption for the table and state the model fit such as pseudo-R-squared, define the abbreviation you used for the reference groups, and the sample size.

**Response#33:** Thank you for your feedback. We have taken your suggestions into account and made revisions to our manuscript. We have provided a caption for the table, and have added information on the model fit in the **table 4**. We also defined any abbreviations we have used for the reference groups, and mentioned the sample size.

**Comment#34:** Line 302 Please consider replacing “being overweight or obese reduce the odds” with “not underweight was associated with reduced odds.” It seems incorrect to specify overweight and obesity when normal weight is also associated with reduced odds.

**Response#34:** We have rewritten the statement as per your suggestion. We have removed the phrase “being overweight or obese reduce the odds” and replaced it with- *“not underweight was associated with reduced odds*”. Now the modified section reads- “*Conversely, higher education, residing in the north-eastern region, and not being underweight were associated with reduced odds of anaemia.”* **[Lines 373-375]**

**Comment#35:** Line 315 Wealthier households may also have a superior sanitation infrastructure, which may decrease infectious disease rates.

**Response#35**: Thank you for your suggestion. I acknowledge that wealthier households may have a superior sanitation infrastructure, which may decrease infectious disease rates. We have included it in our revised discussion- *“Additionally, wealthier households may have superior sanitation infrastructure, which can help reduce infectious disease rates associated with anaemia [60,61]*”. **[lines 396-398]** and have provided references.

**Comment#36:** Line 333 Diversified diets are indeed protective against micronutrient deficiencies, but I am not following your idea of fruits, vegetables, and fish are the ones that help prevent anaemia. These foods are unlikely to provide significant amounts of iron. Please explain how these foods are specifically beneficial. Alternatively, revise this section appropriately.

**Response#36:** Thank you for your valuable feedback. We have revised the section based on your suggestion. We have replaced the line "This is likely due to the diverse and nutritious diet that is traditionally consumed in these states, which includes a variety of fruits, vegetables, and fish" with the following:

*“This is likely due to the diverse and nutritious diet that is traditionally consumed in these states. The culture of the north-eastern states of India may also play a role, with many people following a traditional diet that emphasizes locally-grown and seasonal foods [66,67]. Research revealed Bao or red rice, grown by local farmers of this region has unique nutritional value and is rich in iron, and other micronutrients [68]. This rice is very popular among northeastern people. According to National Sample Survey 2011-2012 (68th round) the red meat consumption (more than 20 grams/day) among the northeastern states was highest in the country. These are among some of the factors that may have contributed to the relatively low rates of anaemia in some of the northeastern states of India.”* **[Lines 417-425]**

We believe that these modifications provide a clearer explanation of the specific dietary factors that could have contributed to the lower rates of anaemia in the north-eastern states of India. Thank you for bringing this to our attention, and we appreciate your contribution to the improvement of our manuscript.

**Comment#37:** Line 345 I would be cautious not to recommend iron supplements because they can be harmful to individuals in the presence of infections. I appreciate that you specify, “as prescribed by their healthcare provider” here.

**Response#37:** Thank you for this suggestion. Upon reanalysing the data and conducting logistic regression modelling, we discovered that the variable for pregnancy and breastfeeding status became insignificant after accounting for other covariates. Consequently, we have made the decision to remove this section from the revised manuscript.

**Comment#38:** Line 361 12 gm% seems like a strange unit. Please check.

**Response#38:** Thank you for highlighting this issue. We have corrected the unit. It is now 12 g/dl.

**Comment#39:** Line 376-377 The sentence is incomplete.

**Response#39:** Thank you for pointing this out. We have rewritten the text to convey our thoughts clearly. The modified sentence is- “*Despite these programs, the anaemia level among women in India, particularly among adolescent women of reproductive age the anaemia is rapidly increasing.”* **[lines 458-459]**

**Comment#40:** Line 386 Around here, you might add a discussion on infections and other lifestyle changes not addressed by these programs as factors behind the increased prevalence.

**Response#40:** Thank you for your valuable comment. We have added a discussion on infections and other lifestyle changes as factors that may contribute to the increased prevalence of anaemia, in addition to the existing government programs **[see lines 449-457].** The revised section now reads as follows:

“…*However, it is important to note that while these programs have made significant contributions to addressing anaemia, there are other factors that may influence the prevalence of anaemia, including infections and lifestyle changes that are not specifically targeted by these programs. Infections, such as parasitic infections and other infectious diseases, can contribute to anaemia by causing blood loss or impairing the body's ability to absorb and utilize iron [74]. Additionally, several lifestyle-like changes in dietary patterns, may contribute to an increased prevalence of anaemia. These factors, if not adequately addressed, can undermine the effectiveness of the existing programs and contribute to the persistence of anaemia in certain populations.”*

**Comment#41:** Line 388 You mention that the study highlights “the interplay of various factors” but to me study has not yet done this. The study has identified factors that are associated with anaemia and you have discussed them individually but very little of the interplay. Please consider editing the sentences with this word. Better yet would be to incorporate the notion of interplay in your conceptual framework and statistical modeling so that you can address how factors may overlap, mediate, or moderate the effect of other factors to give a holistic picture. (This relates to my comments about the issues in your current conceptual framework).

**Response#41:** Thank you for your comment regarding the mention of the "interplay of various factors" in our study. We appreciate your perspective and feedback. Upon reviewing your comment, we understand that you were expecting us to delve deeper into the interplay between the identified factors associated with anaemia.

We appreciate your suggestion of incorporating the notion of interplay in our conceptual framework and statistical modelling. However, given our study's scope and research questions, we believe that it would not be feasible in the current study to go beyond the current analysis and attempt to explore the interplay between factors. Therefore, we have focused on presenting our findings on the individual associations of different factors with anaemia, as was the aim of our study.

Nevertheless, keeping your suggestion in mind, we have made sure to avoid using the term "interplay" in our revised manuscript.

**Comment#42:** Line 393 “women who had children … have lower odds. This contradicts your results reported in the logistic regression table – elevated odds. Please double check.
**Response#42:** Thank you for pointing this out. We have revised the text according the results in the regression table.

**Comment#43:** Conclusion paragraph – please consider editing the words “interplay” and “affecting” (for the reason explained above).

**Response#43:** Thank you for this comment. We have removed the terms “impact”, “affect”, or “interplay” from the modified manuscript.

**Reviewer 2**

Comment#1: This is an interesting and thorough study looking at risk factors for anaemia in India. The main strength of this study is the large sample size but not enough time is spent discussing the dataset or how the variables are defined or collected. More time needs to be spent in the main text **describing the sample,** **the sampling method, and data collection methods**. Likewise, some of the **variables are ill defined**. The **introduction is very long** and words could easily be saved here for them to be used in the methods section. **The information on the adjusted variables could also perhaps be added into a supplementary table.** All variables need to be better defined, but these key ones need to be for the reader to appreciate the manuscript fully:

**Response#1:** Thank you for your feedback on our study. We appreciate your comments and suggestions regarding the dataset, variable definitions, and data collection methods. We agree that providing more information about the dataset and variables would enhance the clarity and understanding of our study.

In response to your suggestions, we have made several revisions in the manuscript to address these concerns. We have dedicated more space in the methods section to describe the sample, sampling method, and data collection methods in greater detail. This have provided a clearer understanding of the study population and the methods used to collect the data (see **the data source, and variables section of the manuscript, lines 134-205**).

Additionally, we have taken your feedback into account regarding the need for better-defined variables. We have carefully reviewed and revised the definitions of all variables to ensure that they are accurately described and easily understood by the readers. We believe these improvements will contribute to a better appreciation of our manuscript (see **Table 1**: **Description of independent variables considered for the study).**

Thank you again for your valuable feedback. We have made the necessary revisions to address your concerns and improve the quality and clarity of our manuscript.

**Comment#2:** Were all independent variables taken from the NFHS-4 and NFHS-5 surveys?

**Response#2:** Yes, all independent variables were taken from the NFHS-4 and NFHS-5 surveys.

**Comment#3:** If different categories of anaemia are being diagnoses (mild, severe, moderate) then the dependent variable is not dichotomous, it is categorical. It needs to be made clear if all 3 categories were dichotomized.

**Response#3:** Thank you for your comment. We have modified the dependent variable section to better convey our meaning **[see lines- 169-182].**

In simpler terms, the NFHS-5 dataset, the level of anaemia is presented as an ordinal variable with four categories: no anaemia, mild anaemia, moderate anaemia, and severe anaemia. To analyze the association between anaemia and various factors among reproductive women, we created a binary outcome variable from the four categories of anaemia. Women with mild, moderate, or severe anaemia were considered "to have anaemia" and were coded as "1". Women with no anaemia were coded as "0" or "women do not have anaemia". This approach allowed us to categorize women into two groups based on their anaemia status: anaemic and non-anaemic. We appreciate your feedback, as it helped us clarify the categorization of the dependent variable in our study.

**Comment#4:** How was household wealth index defined? The categories ‘poorest’, ’poor’,’ middle’, ’richer’, ’richest’ are not very informative. Was the sample split into quintiles based on wealth or were national level scales used?

**Response#4**: Household wealth index was determined based on household scores assigned using principal component analysis. These scores were calculated based on various indicators, such as consumer goods owned and housing characteristics like toilet facilities, flooring materials, and source of drinking water. To determine the national wealth quintiles, the scores assigned to each household member were ranked and then divided into five categories: 'poorest', 'poorer', 'middle', 'richer', and 'richest'. Each category contained 20 percent of the population, and these categories were used as cutoffs to classify households into different wealth quintiles. It is important to note that the wealth index used in our study was derived from the NFHS-5 dataset, and was not created by the authors.

In the NFHS, the sample was divided into quintiles based on wealth, determined by indicators such as consumer goods owned and housing characteristics (e.g., toilet facilities, flooring materials, source of drinking water). These factors were used to calculate a household wealth factor scores using principal component analysis, allowing for classification of households into 'poorest', 'poorer', 'middle', 'richer', and 'richest' quintiles.

**Comment#5:** What is a traditional method of contraception?

**Response#5:** Thank you for your comment. We appreciate your question regarding the term "traditional method of contraception." In our study, the term "traditional method of contraception" refers to specific contraceptive practices that have been used historically and are based on methods such as periodic abstinence, prolonged abstinence, withdrawal, or other traditional practices.

We have added a brief definition of traditional method in **Table 1,** which reads- *“Use of periodic abstinence, prolonged abstinence, withdrawal, or other traditional methods were considered as 'traditional methods' of contraception (coded as 1).”*

**Comment#6:** What BMI cut-offs were used? Were they the south Asian specific cut-offs?

**Response#6:** our paper we have considered World Health Organization (WHO) defined BMI classification. It is defined as a person’s weight in kilograms divided by the square of the person’s height in meters (kg/m2). Body mass index is divided into four categories: 'underweight' (<18.5 kg/m2) (coded as 1), 'normal' (18.5–24.99 kg/m2) (coded as 1), 'overweight' (25–32 kg/m2) (coded as 2), and obese (> 32 kg/m2) (coded as 3).

You can find the details of the BMI categories and their corresponding codes in **Table 1** of our manuscript. If you have any further questions or require additional clarification, please let us know.

**Comment#7:** It is also very important that a comment be made regarding the term ‘backwards class’. Although this term is used by the Indian census it could be seen as discriminatory. This manuscript below uses the term and then has a footnote describing this which could be good to use for guidance. <https://www.frontiersin.org/articles/10.3389/fpsyg.2017.00487/full>

**Response#7:** Thank you for bringing up the issue regarding the term "backward class" and providing the manuscript as a reference. We have taken your feedback into consideration and made the necessary changes to address this concern.

As per your suggestion in **Table 1**, where the independent variables are described, we have added a note to clarify the usage of the term "backward." The note now states: "*The term 'backward' is used in the context of the official classification of caste groups in India, which is recognized by the Constitution of India. The term 'backward' refers to historically disadvantaged groups in India, that are considered socially and educationally disadvantaged. The term has been officially recognized by the Indian government as part of its affirmative action policies to address historical social and economic inequalities in the country. The term 'backward' in this context does not imply a value judgment, but rather refers to the historical social and economic disadvantages that these groups have faced."*

We appreciate your feedback and suggestions, and we have taken steps to ensure that the terminology used in the manuscript is appropriately clarified.

**Comment#8:** Statistical values in text are not adequately described. Using the words ‘around’, ‘large majority’, ‘a little over’ and ‘over’ should be avoided and actual numbers used. Percent also does not need to be spelt out. Using terms like ‘every four out of five women’ (line 218) should be replaced with 80% or a ratio. Overall, the first paragraph of the results is hard to read as % values, ratios and written phrases are all used to describe proportions. It should be consistent.

**Response#8:** Thank you for your suggestion. We have mentioned the exact % values to describe proportions.

**Comment#9:** It is also not clear what a weighted % means in Table 1.

**Response#9:** Thank you for your feedback. We appreciate your comment regarding the term "weighted %" in Table 1. We apologize for any confusion caused. In survey research, weighted percentages are used to account for the complex sampling design and ensure that the results accurately represent the population being studied. The weights adjust the contribution of each case in the sample, taking into account factors such as the probability of selection and differential response rates across subgroups.

To address your concern and make it clearer for all readers, we have made revisions to our manuscript. In the revised version, we have replaced the term "weighted %" with "%" in **Table 1 and Table 2**. Additionally, we have added a footnote to these tables explaining that all percentages have been calculated using women's national weight provided in the NFHS (National Family Health Survey) dataset.

These changes aim to improve the clarity and understanding of the presented data. Thank you for bringing this to our attention, and please feel free to reach out if you have any further questions or suggestions.

**Comment#10:** The English language (spelling, grammar and sentence structure) needs to be improved prior to publication.

**Response#10:** Thank you for your feedback on our manuscript. We appreciate your comment regarding the need to improve the English language (spelling, grammar, and sentence structure) prior to publication. We apologize for any issues with language quality in our manuscript. We take your feedback seriously and tried our best to improve the clarity and coherence of our writing.

**Minor comments**

**Abstract**

**Comment#11:** Around 54-59% of women affected in the abstract is not scientific enough.

**Response#11:** Thank you for your comment. We have revised the text in the abstract to provide more scientific information regarding the prevalence of anaemia among adolescent women. The modified line now reads: "*The prevalence of anaemia among adolescent women in India has increased from 54.2% (99% CI: 53.6-54.8) in NFHS-4 to 58.9% (99% CI: 58.3-59.5) in NFHS-5."* **[see lines 37-38]**

We appreciate your feedback and contribution to improving the scientific accuracy of the manuscript.

**Comment#12:** As the manuscript is in relation to changes over time more statistics about these temporal changes need to be put in the abstract

**Response#12:** Thank you for pointing out the need for more statistics related to temporal changes in the abstract. Despite the 300-word limit, we have revised the abstract to include additional statistics that highlight the changes in anaemia prevalence over time. Thank you for your input, and we hope the revised version meets your expectations.

**Comment#13:** It is not clear from the abstract if nutrition, parity, breastfeeding status, place of residence and SES condition are statistically significantly associated with anaemia.

**Response#13:** Thank you for raising this concern. In the modified manuscript we have added the value of adjusted odds ratio, and 99% CI to make it clear that these factors have statistically significantly association with anaemia. The modified sentences are- *“Several factors were found associated with anaemia, including having more than one child (AOR: 1.33, 99% CI: 1.16-1.51), having no education (AOR: 1.25, 99% CI: 1.16-1.34), belonging to Scheduled Tribes (AOR: 1.47, 99% CI: 1.40-1.53), being in the lowest wealth quintile (AOR: 1.17, 99% CI: 1.12-1.23), year of survey (AOR: 1.26, 99% CI: 1.23-1.29), and being underweight (AOR: 1.10, 99% CI: 1.07-1.12***).” [Lines 44-48]**

**Introduction

Comment#14:** It is not clear from the introduction how anaemia can lead to death – this may be helpful for readers less familiar with the condition

**Response#14**: Thank you for raising this issue. After careful consideration and further research, we have made the necessary modifications to provide a clearer explanation of the relationship between anaemia and mortality in the introduction.

In the revised manuscript, we have removed the statement that "anaemia is responsible for approximately 1.62 million deaths globally each year" as it may have been misleading. Instead, we have focused on presenting accurate information regarding the impact of anaemia on disability-adjusted life years (DALYs).

Specifically, we highlight that anaemia contributes significantly to years of healthy life lost due to disability (YLDs), with the highest burden observed in regions such as South Asia, Western Sub-Saharan Africa, and Eastern Sub-Saharan Africa. We hope this clarifies the issue and provides a better understanding for our readers.

Now in revised manuscript we have clearly written- *“Globally anaemia is responsible for approximately 50.3 million years of healthy life lost due to disability (YLDs) in 2019 with highest YLDs attributable to anaemia coming from South Asia, Western Sub-Saharan Africa and Eastern Sub-Saharan Africa [3].”* **[Lines 64-66]**

Thank you once again for bringing this to our attention, and please let us know if you have any further questions or suggestions.

**Comment#15:** The introduction is interesting but quite long and repetitive. Some of the statistics could be taken out and the same overall message of the importance of anaemia be portrayed. (Notably sections from lines 70-87, 106-115).

**Response#15:** Thank you for your feedback. We appreciate your suggestion to streamline the introduction and avoid repetition. Based on your suggestion, we have revised the introduction to present the importance of anaemia more concisely while maintaining the overall message. We believe that the latest version of the introduction is now improved and presents the importance of anaemia in a concise and cohesive manner. We have taken into account the length, repetition, and logical flow to create the best possible introduction for our study **[See lines 54-131].**

Thank you for bringing this to our attention. We have put significant effort into revising and refining the introduction based on your feedback and suggestions, and we hope the modified introduction better meets your expectations.

**Comment#16:** Line 87 needs a reference

**Response#16:** We have added suitable references to line no 87.

**Methods**

**Comment#17:** More information on the sampling procedure would be nice in the main text. Was every adult approached or was it a targeted sample?

**Response#17:** Thank you for your feedback. In the revised manuscript we have clearly mentioned the sampling procedure in detail. The NFHS sample design is usually two-stage probability samples drawn from an existing sample frame, generally the most recent census frame.

Not, not every adult in the sampled households was interviewed. For example, in both the NFHS, the women’s sample was limited to females aged 15-49 and men’s sample was limited to females aged 15-54 only.

**Comment#18:** It may be nice to provide some more information into similarities and differences between datasets

**Response#18:** Thank you for your feedback. As per your suggestion we have added a brief paragraph stating the similarities, sample procedures, and differences between the datasets.

**Comment#19:** What type of blood sample (plasma or serum)? Were they fasted (lines 158-159)

**Response#19:** We have reviewed the available information on the DHS program website and in the NFHS reports and manuals and found that neither source provides clear information on the type of blood samples collected. However, it is mentioned that the anaemia test was done on women aged 15-49 years through a finger prick. The information about whether they were fasted or not is also provided in the NFHS reports and manuals.

**Comment#20:** Who was eligible? (Lines 159-160)

**Response#20:** The eligible population for the survey consisted of women aged 15-49 years who are usual household residents or are visitors that have stayed in the house the night before the household interview took place.

**Comment#21:** Dietary habit should be changed in vegetarianism as other dietary habits exist.

**Response#21:** Thank you for this suggestion, we have modified the name of the variable ‘dietary habit’ to ‘vegetarianism’, in the revised manuscript.

**Results**

**Comment#22:** The state-wide prevalence trends appears to come out of nowhere. It should be mentioned in the methods that this was going to be done so that the manuscript is clearer for the reader to follow.

**Response#22:** Thank you for your comment. We appreciate your feedback and agree that providing clarity in the manuscript is important. In response to your suggestion, we have made revisions to the methodology section of our manuscript to explicitly mention the state-wide prevalence trends analysis and the rationale behind it.

Modified text now reads*- “To assess the temporal change in anaemia prevalence during the study period, we initially calculated the prevalence of anaemia at the national level. However, as national-level data may obscure regional variations, we proceeded to calculate the prevalence of anaemia for each individual state during both time periods. This allowed us to analyse the state-level changes in anaemia prevalence between 2015-16 and 2019-21. By examining the state-level data, we could gain insights into any variations or trends in anaemia prevalence at a more granular level”.* **[see lines 220-225]**

**Discussion**
**Comment#23:** Line 376 is not a full sentence

**Response#23:** We have completed the sentence in our revised text.

**Tables**
**Comment#24:** Adding in the years the NFHS were taken in the table may be helpful to the reader to appreciate why some associations were lost.

**Response#24:** We have now mentioned the year in **Table 4.**

**Comment#25:** For the reference group in table 3 it may be clearer to put the OR as 1 in the table as typically seen.

**Response#25:** Thank you for your suggestion. In **table 4,** to make our reference categories clearer we have put the OR as 1 for each reference category.

We sincerely appreciate the insightful suggestions and comments provided by the anonymous reviewers. Their valuable input has made significant improvements throughout the manuscript. In response to their feedback, we have taken diligent measures to enhance the abstract, introduction, and data and methods sections. These improvements include restructuring the content, ensuring clarity of objectives, and providing comprehensive details on the study design and analysis. Furthermore, we have re-conducted the data analysis to address specific concerns raised by the reviewers, thereby bolstering the validity of our findings. As a result of these comprehensive revisions, we believe that the revised manuscript is now of higher quality and better suited for publication in your esteemed journal. We express our gratitude for the opportunity to submit our work and eagerly anticipate the possibility of sharing our research with your discerning readership.

Sincerely yours,

Mahashweta Chakrabarty

Aditya Singh

Shivani Singh

Sourav Chowdhury
